# "The Hospital" Nursing Section

**Published:** 1906-09-22

**Authors:** 


### The Hospital.

### Nursing Section.

Contributions for "THE HOSPITAL," should be addressed to the EDITOR, "THE HOSPITAL" NURSING SECTION, 28 & 29 Southampton Street, Strand, London, W.C.

No. 1,044.—VOL. XL. SATURDAY, SEPTEMBER 22, 1906.

### Notes on News from the Nursing World.

### THE DUCHESS OF CONNAUGHT AND THE MOTOR ACCIDENT.

IT has been stated in the lay papers that the Duchess of Connaught acted as nurse in the case of the boy who was fatally injured by the motor-car in which her Royal Highness and her daughters were being driven from Bagshot to London last week. As a matter of fact, the Duchess, who from the time the accident occurred manifested the most kindly sympathy for the unfortunate lad, simply stayed in the surgery of a local medical man where he was taken for some time, and showed her sympathy for the sufferer. She could not have manifested more attention if he had been one of her own children, and the catastrophe, instead of being absolutely unpreventable, had been due to carelessness on the part of her chauffeur. But the nursing of the boy was done at the Hounslow Cottage Hospital—where he was conveyed in the police ambulance—under the auspices of the matron, Miss Jacobs. Although his life could not be saved, it is pleasing to know that everything possible was done for him in the Hounslow Hospital.

### SISTERS AND ORDERLIES.

THE latest suggestion of the opponents of the employment of female nurses in the Army is that pressure should be brought to bear upon Mr. Haldane and the Army Council in order to obtain an investigation with a view to a reduction of expenditure. The Secretary for War may be trusted to resist, or to yield, to this pressure as he thinks desirable in the interests of the country. But, meanwhile, we do not think that it is worth while to pursue the question further in our correspondence column beyond the insertion to-day of a letter which speaks for itself. This communication and others received by us do not deal with any points which have not already been discussed, and do not affect the issue in either direction. It is quite hopeless to try and convince members or ex-members of the Royal Army Medical Corps who stigmatise the employment of Army sisters as a "sore," or who affirm that "the good, just sisters average about one in each first-class military hospital," of the injustice of their assertions; and the admirable work known to have been done by the latter forms the best reply that can be given to their critics. If an inquiry takes place we are confident that the result will not be to strengthen the hands of those who are eager to attribute any possible defects of the system to a policy which has raised the standard of nursing from inefficiency to efficiency.

### POOR-LAW NURSES AND REGISTRATION.

IN the report of the Parliamentary Committee of the Council of the Poor-law Unions Association on the measures introduced into Parliament this year affecting Poor-law administration reference was made to the Nurses Registration Bills. Objection is taken to these measures on the ground that there is nothing in them to prevent the professed Central Board from declining to recognise Poor-law infirmaries as nursing schools, and that if they did so decline girls would probably not enter the Poor-law Nursing Service as probationers, knowing that the training they would receive in such service would not qualify them to be registered as nurses, and thus enable them to obtain employment in the best class of private nursing. We quite agree with the view that no Registration Bill can be fair to Poor-law nurses which does not contain full provision for the recognition of acknowledged training schools attached to Poor-law infirmaries; and that in the event of legislation the definition of what is to constitute a recognised training school should be such that persons trained in Poor-law schools would be in a position to claim the right of sitting for examinations to be conducted by the Central Board. Although we do not believe that the Government will take up the question of registration next session, we think that the Poor Law Unions Association will do well, in the interests of their large and important schools, to keep this matter to the front. At the same time, warned by the refusal of the Central Midwives Board to recognise so many of their infirmaries as fulfilling the conditions of training for midwives, the Local Government Board may be relied upon to take care that a similar state of affairs does not arise in respect of the recognition of their nurse-training schools.

### MASQUERADING IN NURSES' UNIFORM.

LAST week we published a letter from a correspondent complaining that the nurses in a certain town habitually lend their uniforms every year to ladies to collect money for the hospital. Our correspondent expressed her surprise and indignation at the indiscretion of the nurses in lending their uniforms for the purpose. But we are still more astonished that the authorities of a hospital of the importance indicated should permit such a course to be pursued. Under no circumstances whatever can masquerading in nurses' uniforms be excused, and women have no more justification for using it in the interests of charity than laymen would have justification in dressing up in clerical attire, or civilians in donning military uniform. So long as the authorities of the hospital in the town referred to are privy to the practice complained of, they are doing their best to discredit the position of their nurses.

### A LOCAL GOVERNMENT BOARD INSPECTOR ON MALE NURSES.

At Totnes last week the Guardians discussed the case of a male attendant who had been accused of insolence and abuse to the superintendent nurse. He was allowed to appear before them in order to vindicate himself from these charges; but he admitted that he had refused to obey the instructions of the superintendent nurse. Subsequently, Mr. Preston Thomas, Local Government Board Inspector, who was present on the occasion, stated that in the forty-seven unions in the West of England there were only three or four male nurses employed, and said that where they were in small workhouses there were often quarrels or scandals. He confessed that he was rather against the system and in favour of a staff consisting entirely of females. As to imbeciles, he did not think that they should be in workhouses, except in separate wards under their own attendants. Mr. Thomas was pressed to concede that in existing circumstances it might be desirable to have a male nurse in a workhouse, but he declined to modify the opinion he had expressed.

### THE POSITION IN ITALY.

The particulars which an Italian nurse, whose contribution has been translated, gives in our columns to-day of the nursing done in Italy by the natives shows that the rate of progress continues to be very slow. An attempt, our correspondent states, was made a few years ago in Florence and in Rome by an English lady to bring about the training of girls of the middle-classes in hospitals, but it did not meet with much success; and now, apparently, native nursing is confined to nuns and to girls who have little or no education—some of them being taken from work in the fields or in kitchens. No doubt the explanation is given in the last sentence of the article, and may be found in the fact that Italians generally are imbued with the idea that nursing is simply a synonym for the religious life. Until it is brought home to them that there is nothing derogatory to a woman of any class pursuing the calling of a nurse, even though she does not pledge herself to renounce the vanities of the world, the position is not likely to improve.

### A CANADIAN MATRON AND HER NURSES.

Some very admirable advice is given by Miss F. Wilson, lady superintendent of Winnipeg General Hospital, to her nurses in one of her class-papers. She tells them not to lean against the beds; to get in the habit of never touching a bed when walking or standing about the wards; to form habits of walking and talking quietly. She urges them not to walk on tiptoe, because "it is very distressing to listen to"; to look cheerful and speak cheerfully; to be thoughtful to the patients' friends, as well as to pay little unselfish attentions to the patients; and to think of the trouble they are in and of the misery and heartaches they are enduring. The night nurse, she points out, has "such an opportunity of being a nurse"; she can see that the feet of the patient are warm, perhaps give a hot sponge, or rub the weariness of lying out of the back, or put cold cloths on the head, or speak in a gentle soothing voice. Miss Wilson's remarks on economy and care of hospital property may be commended to the attention of nurses who, perhaps, lightly regard this matter; and she instances in particular the nuisance of gauze for handkerchiefs, for padding crutches, for cleaning instruments, and for washing basins in bath-rooms, as well as the waste of it in dressings. We are afraid that a good deal of this misuse and waste goes on in our own hospitals.

### IMPROVEMENTS AT CARNARVON.

Although the Carnarvon Guardians at their last meeting endorsed the report of their own committee that a series of complaints made by the workhouse nurses were "trivial," and "bordering upon being frivolous," certain changes were recommended by the Committee and have been adopted by the Guardians. which go far to justify the representations of the nurses. The manner in which the food is placed on the table is to be improved, the meat and vegetables are to be served in separate dishes and conveyed in a small covered van from the kitchen to the infirmary. A maid is also to be employed exclusively in the infirmary, so that the table may be properly laid for the nurses and that they may be waited upon during meals. These are not only distinct improvements, but afford the clearest possible proof that there was substantial cause for dissatisfaction with the previous arrangements. For the rest, we think that the appeal of the Guardians to the nurses on the one side and the master and matron on the other to avoid friction by the exercise of patience and tact should be responded to. So long as the nurses have to take instructions from the master and matron they must necessarily recognise their authority; but even under this condition of affairs it should not be impossible for all parties to live in peace.

### AN EXPERIMENT THAT FAILED.

In order to save expense, we presume, the Frome Guardians recently arranged that the night nursing in the Workhouse Infirmary should be comprised in a midnight visit to all the wards by the assistant nurse. The result, however, as reported at a meeting of the Guardians last week, has been that the head nurse has frequently had to get up to attend cases beyond the assistant's care, while the assistant nurse has had to be released from duty every afternoon when her rest had been broken into at night. It was admitted that the experiment had failed, and the question of a new arrangement was referred to a committee. The only proper course is, obviously, to have a qualified nurse always on duty, and any makeshift must, in the long run, break down.

### AN INCIDENT AT AN INQUEST.

At an inquest last week in Glamorganshire the question arose as to how the deceased, who was an invalid under the charge of a nurse, had obtained the four and a half grains of chloral which caused his death. The nurse stated that she knew nothing about it, and that neither she nor her patient possessed any drugs. In summing-up the Coroner said that the jury must either disregard the evidence of the medical experts and think they had made a mistake, or believe that the nurse had not told the whole truth. The nurse burst into tears, and was again visibly affected when the verdict was given, though it did not in any way reflect upon her. If there was anything more to tell it is a pity, for her own sake, that she did not tell it, because, as the Coroner said, she could in no way benefit by bringing about the patient's death.

### MEDICAL OFFICER AND SUPERINTENDENT NURSE.

The medical officer of Guildford Workhouse has applied to the Guardians for an honorarium of ten guineas on rather unusual grounds. The late superintendent nurse was away from her post for some time owing to illness. The medical officer protests that in any case he should not charge for attendance upon members of the staff, but he claims the honorarium in question on account of "the extra strain and work imposed upon him by the absence of the superintendent nurse." The matter has been referred to the House Committee, and their decision will be awaited with interest. A temporary superintendent nurse was on duty for a considerable period.

### APPOINTMENTS TO THE MILITARY NURSING SERVICE.

We are officially informed that Miss C. Mackay, sister, has been transferred to the Military Hospital, Shorncliffe, from the Queen Alexandra Military Hospital, Millbank, and that Miss E. Foster, sister, has been transferred to the Connaught Hospital, Aldershot, from the Royal Victoria Hospital, Netley. Other appointments in Queen Alexandra's Imperial Military Nursing Service are that of Miss M. McKenna as staff nurse to the Queen Alexandra Military Hospital, Millbank, and that of Miss M. Tedman as staff nurse to the Royal Herbert Hospital, Woolwich. Miss J. G. Powell, Miss E. M. Denne, Miss M. Walker, and Miss L. E. Mackay, sisters, have been ordered to hold themselves in readiness for service abroad.

### VICTORIAN NURSES AND FINANCE.

The financial statement of the Royal Victorian Trained Nurses' Association for the year ending June 30 shows that the members' subscriptions amounted to £426 8s. 9d., and the general expenses to £235 2s. 10d. There are balances at the Commercial Bank of £165 0s. 7d., and at the Savings Bank of £212 6s. 1d. The sum of £50 was transferred to the Benevolent Fund, and £100 to the Committee, who are responsible for the publication of *Una*, the organ of the Association. A paragraph in the annual report is headed, "Unfinancial Members," and has reference to the minority who are two or three years in arrears with their subscriptions, though they have not applied for any exemption. This, of course, is not fair to the majority, and, we agree with the Council, supplies a sufficient reason for removing the names of the offenders from the register of a purely voluntary body.

### LECTURES TO MIDWIVES.

The lectures under the auspices of the London County Council to practising midwives (certificated and *bona fide*) will be resumed this autumn, and further classes will beformed all over London if a sufficient number of midwives signify their desire to attend. These lectures are practical, and are delivered by medical men, and should be appreciated by both trained and untrained midwives who desire to keep themselves in touch with modern methods. They will also be helpful in explaining the requirements of the Midwives Act and the rules of the Central Midwives Board. The fee is only a shilling for the course of twenty lectures. Miss Gill, Secretary of the Association for Promoting the Training and Supply of Midwives, Dacre House, Dean Farrar Street, Westminster, will notify to any midwives who signify their wish to attend the classes the nearest centre where lectures will be given.

### A PHOTOGRAPH FOR NURSES

The issue of *Photography* for September 11 contains a reproduction of a clever photograph by John Hepburn, entitled "The Village Nurse." The picture represents a child with bandaged head lying on an improvised couch in one of those untidy cottages which are more dear to the heart of the artist than to that of the nurse. The mother, standing behind the couch, leans over the child, while the nurse, in bonnet and apron, sits in front, medicine-spoon in hand. On the floor in front of her is her open bag, and by her side is a rough stool, on which stand a basin and a bottle of, we imagine, disinfectant. With regard to these details, it is interesting to read what Mr. Frank Selwyn, who writes the article to which the picture is an illustration, says: "I cannot help thinking that the matter of accessories is overdone. Quite half the number might be removed without leaving unsatisfactory blanks in the composition, and with a distinct increase in its concentration. The bag and stool are cases in point." That is the point of view of the photographer, perhaps of the artist; it is not ours, nor, we think, that of any nurse. In our judgment, these details add to the realism of the picture, which in its way resembles the pathetic work of Israels; nor do they, to our eye, in any way distract from its artistic value.

### DISPENSARY TRAINING IN HOSPITALS.

It may be of interest to nurses who propose to take up dispensing to be reminded that for the past thirty years ladies have been trained in the Out-patients' Department of the Birmingham and Midland Hospital for Women, usually six to eight at a time. Miss Blanche Thompson, the dispenser and lady superintendent of the Out-patient Department, has been instructor for twenty years, and has had a successful record at the Apothecaries' Hall examinations.

### SHORT ITEMS.

Messrs. John Wright and Company, of Bristol, will shortly issue a complete set of large midwifery diagrams designed for the use of lecturers to midwives and elementary students by Dr. Victor Bonney. The whole set will be supplied for two guineas, or single sheets at 2s. each.—In the summary of training schools recognised by the Central Midwives Board which we gave last week it should have been stated that the 200 midwifery cases treated at the Birmingham Poor-law Infirmary in twelve months were internal, not external; and the Coombe Lying-in Hospital, Dublin, was printed as the Camp Hospital, Dublin.

### The Nursing Outlook.

"From magnanimity, all fears above;
From nobler recompense, above applause,
Which owes to man's short outlook all its charm."

### SOME ESSENTIALS OF SOUND ADMINISTRATION.

It falls to our lot, in the ordinary course of the year's work, to inspect many institutions. It is frequently noticeable that the ruling authorities, in their desire to promote economy or efficiency, often make grievous mistakes. One constant error in dealing with and judging the quality of the work done by the chief officials is an absence of appreciation of the principles which must underlie the best administration of a hospital and training school for nurses. Committees frequently fail because they are not practical, and because no means are at present forthcoming of which members, or intending members, can avail themselves with a view to acquire a practical knowledge of the duties which devolve upon them in such a capacity. The School of Economics, or The Hospitals Association, or the King's Fund, or perhaps the Hospital Officers' Society, might institute a course of lectures on sound administration, and the kind of information essential for honorary managers of hospitals of all kinds.

In a hospital where probationers are trained, where a visit of inspection may reveal an absence of efficient supervision or the disregard of small matters which materially influence the comfort of the working staff, it is fair to assume that the chief official has failed to grasp the importance of so organising the school under her charge as to make it the centre of the life of all who may enter its walls. In a great business success is known to depend largely upon the efficiency of each individual worker. Such success is only attainable through an organisation which includes a trained band of overseers whose business it is to know the capacity and the stage of advancement in knowledge of each individual worker. Under such a system everybody grows to be happy and contented, for it secures promotion in accordance with capacity and qualification, based upon accurate knowledge of the individuals who collectively constitute the workers in such a hive of industry. In the same way to attain the maximum of success, a nurse-training school must be so organised as to give every probationer the fullest opportunity to develop every quality of value she may possess. The system should afford her a guarantee that in due course, when she has completed her training, her school will provide for her a position and promotion which will do justice to her attainments, if they may not even properly satisfy her ambitions.

In county and smaller hospitals especially, and in some of the larger hospitals, too, it is still the practice to encourage strangers from outside the school to occupy the position of sisters, and even higher offices too. Such a plan is foredoomed to failure, and deserves to fail. Is it reasonable to suppose that the authorities of a well organised training school will suffer the loss of the services of some of their ablest and best pupils in order to let them take office in another institution? In fact the main justification which hospital committees have at the moment for the enormous increase in the cost of a hospital bed nowadays, owing to the development in nurse training and the huge outlays on nursing homes and plant, is the fact, that, through the school they are able to provide the wards with the trained nurses of the highest qualifications which the requirements of the patients and the medical staff demand. Wherever then a hospital committee finds that they are encouraging and training probationers as nurses, whilst they are continuously asked to sanction the appointment of outsiders to the posts of head nurse, sister, or night superintendent, they may rest assured that they have not in fact a well organised training school for nurses. Indeed men of business who regard their position as committeemen as offices of trust must have grave doubts, in such circumstances, if they face the matter fairly and squarely, as to whether they are justified in sanctioning any further expenditure upon such a nurse-training school at all, so long as it fails to supply the hospital with trained nurses in adequate numbers.

And the moral of such a condition of affairs is obvious too. If a training school cannot supply a sufficiency of capable trained nurses for its own requirements, what justification has its committee to grant certificates of training to probationers who may have spent two or three years within the walls of such an ill-conducted hospital? If the training or the quality of the material is such that the best and most capable nurses are not produced, clearly the whole establishment is a mistake, and the training school, as it must have been wrongly called, should be recognised as a failure and dealt with accordingly. We recently visited a hospital which advertises for probationers and professes to train them as nurses. We found failure written across the face of this establishment by the fact that promotion was denied to the nurse when she had completed her training, and that sisters and head nurses were selected from other institutions—not from the school ranks. Going a little below the surface we were not surprised to learn that the quality of the nursing throughout this establishment left much to desire from both the patients' and the practitioners' point of view.

### Abdominal Surgery.

By Harold Burrows, M.B., F.R.C.S., Assistant Surgeon to the Seamen's Hospital, Greenwich, and to the Bolingbroke Hospital, Wandsworth Common.

### PREPARATION FOR ABDOMINAL SECTION.

There is not space here to enter fully into the subject of how to prepare for an operation. On this account the following remarks must be regarded merely as a collection of hints which, it is hoped, may prove useful to those who already possess a general knowledge of the subject.

In preparing a patient for any operation one of the essential matters is to ensure a thorough clearance of his bowels beforehand. In abdominal cases this is of even greater importance than in others. Often it is well, if time allows, to commence by giving an aperient at bed-time, two days before the operation, to repeat the dose on the following night, and to give an enema on the morning of the day fixed for the operation. But this is not suitable for every case, and an intelligent discretion is very desirable in these matters. It is to be borne in mind that castor oil is not the only good aperient, and some patients object strongly to taking castor oil. In such a case it is a good plan to use the medicine to which the patient is accustomed and the effect of which he is acquainted with. In every case, whatever the methods employed, this preliminary treatment should be so managed as to interfere to the least extent possible with the patient's repose, for a good night's rest before an operation is most beneficial.

If the patient is not too ill he should have a warm bath on the evening before the day of operation. And on the same evening the abdomen must be disinfected and a preparatory dressing applied. The skin must be shaved if necessary, and then well washed with soap and water. A nail-brush ought not to be used for this purpose, as it is apt to excoriate the skin. The best scrubber is a washing-glove that has been previously boiled; this will remove the dirt without making the skin sore, and its use is not uncomfortable to the patient. The soap is then washed off, and ether or turpentine is applied in order to remove the fat from the skin. A piece of lint wrung out of a watery solution of biniodide of mercury (1-1,000) is then spread over the area that has been cleansed, and is covered by a piece of jaconet a little larger in size, and kept in place by a bandage. This compress is not removed until the patient is under the anæsthetic and the operation is about to commence.

A mistake which is made with lamentable frequency is the preparation of too small an area of skin. Not only must the skin of the entire abdomen be disinfected, but also that of the pubes, the upper part of the thighs, the lower part of the chest, and the loins.

Occasionally one other error is made, and this is blistering of the patient's skin. As mentioned in a previous article, the improper use of turpentine stupes, or even the application of simple fomentations which are too hot or which have not been wrung out sufficiently will cause vesication. But blistering may occur even when no hot fomentations or turpentine stupes have been used. In these cases it is often due to applying the antiseptic compress and jaconet mentioned above before the ether or turpentine, used to remove the fat from the skin, has had time to evaporate. Time should be allowed invariably for this evaporation to take place before the lint and jaconet are placed in position.

Now blistering of the skin is a deplorable accident, for asepsis cannot be assured unless the skin is healthy and undamaged. Consequently, if the skin is blistered, either the operation has to be postponed for several days; or, if this be impossible, the patient must be submitted to operation in circumstances which greatly increase the dangers of the procedure. The annoyance and trouble caused by this wretched complication to patient, surgeon, and everyone else concerned may be left to the imagination.

In the case of hysterectomy or of other operations on the female genital organs it is necessary to disinfect the vulva and vagina, in addition to the abdominal wall. If there is any septic discharge from the vagina, antiseptic douches should be used twice or thrice daily for some days previously.

In discussing peritonitis, appendicitis, and other inflammatory conditions in the abdomen much stress has been laid on the part played by micro-organisms derived from the bowel. In view of this it seems not unreasonable to avoid so far as possible the entry of pathological organisms into the alimentary canal. This is to be done, firstly, by keeping the patient's mouth clean, and, secondly, by boiling the milk or other food before he takes it. Of these two, the former is probably the most important, and it is a point that always requires careful attention in abdominal cases.

When a patient is about to be operated on for peritonitis, intestinal obstruction, or other condition attended by vomiting, it is often desirable to wash the stomach out before the operation commences and again after it is concluded. By having the necessary apparatus in readiness valuable time may be saved.

In every case of abdominal section the patient's bladder must be emptied immediately before the operation. Frequently the catheter will be required for this purpose. In pelvic operations it is nearly always a wise measure to use a catheter, for it is only in this way that the required result can be secured with completeness and certainty.

With regard to bandaging after an abdominal operation, a many-tailed bandage is preferable to a simple binder, because with the former it is more easy to obtain an even distribution of firm pressure. Perineal bands are advantageous, especially when the operation wound is in the lower part of the abdomen. I have seen an abdominal wound completely bare on the day after an operation, owing to the dressings having slipped upwards, there being no perineal bands to keep them in place. Such an accident is little short of a catastrophe.

### The Nurses' Clinic.

### BLADDER AND KIDNEY CASES.

To every nurse comes sooner or later the duty of nursing bladder or kidney cases, or both, and we know from experience amongst them with what pain and discomfort these complaints are usually attended. Certain leading symptoms are always present in these cases, and these symptoms may result from a variety of causes. There may be simple cystitis, stone in the bladder, cancer of the bladder, simple or tubercular ulceration of the bladder, kidney disease, stricture of the urethra, growth in the urethra, or perhaps pressure upon the bladder by other organs in the pelvic cavity, pressure caused by abdominal tumours or pregnancy, or even pressure from overloaded bowels. It is not the province of the nurse to diagnose complaints, but she will nurse a case all the better if she understands something of cause and effect as indicated by objective symptoms in disease. Whatever may be the cause of the trouble there are certain important points which the nurse should observe in connection with every case of the kind under her care. She must note the colour of the urine when passed, whether it is passed in very small quantities, even a few drops at a time as in cases of stone in the bladder when micturition is attended by intense pain, or whether an unusual quantity is passed as in diabetes and some aspects of kidney disease, whether it is very pale like hot water as in hysteria, or thick and muddy in appearance as in liver derangements. It is well to remember that certain medicines affect the colour of the urine and that absorption of carbolic into the system has a similar effect. Normal urine is of a pale yellow colour, clear when passed, though it becomes cloudy after standing for some time. The usual quantity passed in twenty-four hours is about 56 ozs., but this amount is capable of great variation even in healthy persons. Excitement of mind or worry frequently cause an increased flow of urine, while feverish conditions of the body will have a reverse effect. A normal bladder will hold about 20 ozs., but different persons differ greatly in the frequency with which evacuation takes place. The urine of gouty patients is heavily charged with uric acid which leaves a reddish stain upon standing. Pus in the urine is easily observed by stringy deposits of an unpleasant odour, but albumen cannot be verified except by testing. Blood in the urine is always a symptom of importance; if bright red in colour it proceeds from the bladder, but if of a smoky tinge it comes from the kidneys. It frequently happens that temporary paralysis of the sphincter muscle of the bladder occurs after an operation or any other shock to the system. This condition may be relieved by the application of hot fomentations over the bladder, but if retention is not relieved in this way, the doctor should be informed without delay. If circumstances permit, a sitz bath often gives relief, while even a change of posture from recumbency to a knee and elbow position is sometimes successful. Ulcertaion of the bladder whether simple or tubercular may exist for a long time without any unusual appearance of the urine, but this condition will always be attended with pain and discomfort and probably at intervals with severe neuralgic pains. It is therefore doubly important to report these symptoms that the cause may be sought for and treatment given. The nurse should know the difference between suppression of urine and mere retention, the former being attended with great danger while the latter is of minor importance and can be relieved by prompt measures. Most nurses in these days learn to test urines, and in this way they may be of great assistance to busy medical men, but this must always be done with care and reported with great accuracy, as much may hang upon the result of urine tests. In every hospital it is an understood thing that bladder and kidney cases require all urine to be measured and the amount charted and a specimen is put up daily for the doctor's inspection. This routine is equally important, if not more so, when the nurse finds herself away from all the associations and appliances to which she has been accustomed during the years of her training. If catheterisation is ordered every precaution must be taken to avoid introducing sepsis into the bladder. The catheter should be either sterilised or soaked in an antiseptic solution for five minutes before use, and after use well cleaned and then kept in 1 in 40 or 60 carbolic lotion. To prevent irritation of the urethra it is usual to dip the end of the catherer in carbolic oil, glycerine, or pure vaseline before introduction. If a sense of chill succeeds the passing of a catheter, the patient's temperature must be taken, a hot drink given, and hot-water bottles placed in the bed, and the fact reported to the doctor if the temperature has risen much above normal, as "catheter fever" is a catastrophe to be avoided by every possible means. Every patient suffering from bladder or kidney trouble should be warmly clad, especially in the neighbourhood of the parts affected, as a chill contracted during the illness or convalescence may mean a serious relapse, with a repetition of all the old symptoms. During the acute stage of the disease, the patient is kept in bed both for warmth and for the benefit of a recumbent position. Instructions will be probably given in the matter of aperients, as it is very important in these cases to avoid constipation. In the absence of special orders, cascara tabloids or liquid cascara sagrada in moderate doses will prove efficacious, as it is desirable to avoid violent purging as well as constipation. The nurse will soon discover how much or how little the patient needs in this direction. While one patient needs six tabloids, another finds that one is quite sufficient for the purpose. The question of diet is usually determined by the doctor, but as a general rule light nourishing food is ordered with a scarcity of meat and abundance of milk. Barley-water, linseed-tea, and all such mucilaginous drinks may be given freely; but alcohol and all stimulating or spicy condiments are to be avoided. In cases of congestion of the kidneys, we know that the uric acid formed in the system cannot be got rid of in the ordinary way, therefore the doctor will probably order special medicines to induce perspiration that the skin may work more actively and carry off the poison which would otherwise accumulate in the body and cause serious trouble. The nurse should at all times note the effect of special medicines, and, in fact, special treatment of all kinds, as upon her report often depends the future treatment of the case : here comes in the great value of the faculty of observation. Nothing is trifling in serious illness, and a nurse who can report accurately is appreciated highly. The care of these cases after operation does not differ very much from any other kind of case, except that if there is any involuntary passing of urine the skin must be well looked after; and the patient kept clean and dry to avoid bed-sores. If the case has been operated on for floating kidney, the bedstead will be probably raised some inches at the foot, so that no strain may be felt in the affected region.

Great care is required in moving the cases in question, so that the tube and dressings may not be disturbed, and for three weeks at least they must neither sit up nor make any movement to help themselves. Patients of this class, indeed

**THE NURSES' CLINIC**—*Continued.*

all who suffer from internal complaints, are apt to become very depressed and need cheerful surroundings and hopeful nurses; so many nerves are involved in these affections that neuralgia always aggravates the original trouble, and low spirits and depression naturally follow. Prolonged rest and assiduous care are very important after operations on the kidney or bladder, and though the patient may feel able to walk, it by no means follows that it is wise or safe to do so. Weakness of the parts will continue for a long time, and if complete recovery is to be hoped for normal habits must be resumed very gradually indeed. Neglect of these precautions may mean invalidism for life, not to mention pain and deprivation of many of the joys of existence. There are few cases more distressing to nurse than elderly male patients who suffer from stricture of the urethra; alleviations such as hot baths if practicable, or hot compresses may give some relief, but frequently are of little avail. Cases of growth in the bladder, or growth or swelling in the urethra cause such extremity of pain and misery both in walking and sitting that nurses need an extra supply of patience and sympathy in dealing with them; there is only one way and that is the way recommended by Florence Nightingale, we must put ourselves in the place of the patient and act towards him as we should wish to be done by were our positions reversed.

### Incidents in a Nurse's Life.

### AN AUSTRALIAN VENTURE.

A telegram was put into my hand on one of the hottest days at the end of the long hot summer. It was from a doctor, whom I did not know, asking me to nurse a case in a township 400 miles inland. The "Australian tired-feeling" was hard on me, but I scribbled on the spare form, "Will come by next train," and then, when the telegraph-boy had gone, wished with all my heart that I had said "No." No one knows but those who have felt a hot land breeze blowing off a thousand bush fires what it makes one feel like—floppy, sticky, depressed, unutterably dry, dry eyes, dry throat, dry nostrils, dry skin, dry everything. The train left in the afternoon and arrived at my destination in the middle of the night. It started by going straight up into the hills, which sounds cool, but, oh! it was hot! Fires were raging in the scrub on all sides, and the rocks, which are running with wet and covered with moss and ferns in the winter, were baked and barren, giving off a great heat. How the train dawdled along! There is only one train a day into the "way back," and it is mail, passenger, newsbearer, luggage train, besides carrying children to and from Government schools all along the line, when it passes near their homes at convenient times. I have seen a little child alone on the side of the line holding up her hand, and the great thundering locomotive answering the gentle signal and stopping, simply to take her up and on to school. Over the hills we came to the wooded country, where camps and little corrugated iron shanties mark the dwellings of timber cutters. There were not so many fires here, as they had been beaten back and other preventions used. By and by we passed isolated farms, generally fruit farms where rows and rows of vines laden with grapes met the eye, or beautiful shining-leafed orange trees with clusters of golden fruit peeping through them. It was getting dusk when we came to the only township of any importance on the line, and had dinner, and when we were well on the way again it was quite dark. I dozed for a time, but presently started up and looked out of the window sniffing the pungent odour of wood burning. And then I saw a bush fire! As far as the eye could reach below were hundreds and thousands of sparks, and little dancing red flames, and big blue flames, and columns and volumes of glowing light, and catherine wheels and squibs and rockets, and, above all was the clear still dusk and the gentle stars.

"Rome burning," remarked someone in a corner. "Terrible good fire for toast," grinned a fat boy. "H'awful waste of good wood," added a maternal-looking lady, evidently just out from the Old Country. I curled myself up in my corner and dreamed. It was getting cooler, and when I reached my destination in the middle of the night it was quite chilly. The doctor met me, much to my surprise and relief, and took me to a boarding-house from where he drove me to my patient next day. It was a very bad case, five miles from the medical man. While I was there I made up my mind to find a cottage in the town and turn it into a private hospital. Case after case I heard of that only needed medical care and nursing to save life—the patient I was nursing being one in point. When it was over I consulted the doctor, who was only too pleased. He was a young Englishman, desperately out of love with his lot, because everything seemed so hopeless; and, besides, he was not used to hot winds and dust and long rides and no one to talk to. "I grow roses," he explained rather piteously, "to save me from going mad. You'd better think before you make up your mind to come to this God-forsaken hole." "Was there plenty of work?" was all I wanted to know, and as he said there was, I came.

More correctly speaking, we came, for I brought my sister—not a nurse—to help. We had no servant, but we turned our dirty four-roomed cottage and two out-houses into a toy hospital; I will not say "model" because I have seen a model hospital in England, and there the sanitary arrangements were not oil drums; the copper and pails were not kerosene tins; the kitchen furniture was not made out of packing cases; and the chicken house was not built by the nurse in charge. We got the landlord to move the kitchen range into one of the outhouses, clean the whole place thoroughly, and put up a back verandah. However, he left the fireplace in the middle of the room for a week, so we moved the bricks, brushed the mortar up, and made the chimney usable ourselves, while we cooked on a camp fire in the yard. In the middle of this our first patient came in—a boy who had put his hand in a chaff cutter. It took two hours under chloroform to patch it up. I had to give the chloroform. Before the operation was barely over in walked the resident magistrate unexpectedly, also a doctor, from his abode fifty miles away. He said that everything was delightful. He has always been our best friend, but, then, we knew him at home. I cannot explain to you in England what "an Englishman" means to us out here. When in England, if an Australian used the expression I used to think it odd, and say "But you are all Englishmen." Now I know the difference.

We have been here three months, and our surgery furniture is all enamelled white, and we have had two emergency abdominal operations, first intention results, our six beds full, and a real nurse on the staff. I wonder if any English nurse would stand it? No servant, only a washerwoman once a week, meals in the outhouse-kitchen, bed on the verandah, or the other outhouse room (which is also dressing-room), and a wet or a sun-baked yard to run across constantly. But we are very happy on the whole, and are busy in off-duty hours, making a garden which promises a glorious result.

### Nursing at the Waterford County Infirmary.

BY AN OCCASIONAL CORRESPONDENT.

In travelling through Ireland one cannot help noticing that the worthy men who built hospitals in bygone days set them, whenever possible, on the top of a hill. This is all the more remarkable because their own houses and castles are mostly situated in sheltered spots, with an eye to warmth and safety, rather than beautiful views.

The County and City Infirmary stands up boldly on a high eminence, looking across a little valley to the towers and steeples of the old city of Waterford. Immediately in front its own grounds slope gently down, emerald green as the rest of the land, enclosed by high walls.

The building, which was altered and renovated in 1897, was erected as a leper hospital in 1785 by John Roberts, the great-grandfather of the present Lord Roberts.

Since the hospital was converted into the County and City Infirmary, many changes have been gradually taking place. Formerly the management, as in some of the Poor Law Infirmaries, was all in the hands of the Matron, but the Committee decided to alter this, and in October last Miss McDowell was appointed Superintendent of Nurses, with full authority in this department. She was trained at Sunderland, and subsequently held the post of sister-in-charge of the Orthopædic Hospital, Dublin. She also served in the South African War.

The hospital, with general and private wards, contains 50 beds. The wards, each with its eight or ten beds, are bright and comfortable, and the theatre is modern, except for the stove, which is now to be done away with and replaced by hot-water pipes.

There are various other improvements still under contemplation, and different accommodation for the nurses—whose bedrooms are close to the wards—would be a distinct advantage.

Eight of the nurses are set aside for private work, being employed in the wards when they are not out at a case. This department, as well as the actual superintendence of the surgical wards and the theatre, is in the hands of the Superintendent of Nurses. But she likes her busy life, and takes a personal interest in the cases, as well as in the nurses. By her kindly intervention a ward on the ground-floor, formerly used for isolation purposes, has been converted into a sitting-room for the nurses, where they have a piano, books and easy-chairs. The superintendent nurse has her own little sitting-room on one side of the dignified entrance hall, and the matron, who superintends the housekeeping, servants and linen, has a similar one on the other side. The resident house-doctor's rooms, and other offices, are also on this floor.

The usual period of three years' training is given, with a certificate on passing the examination at the end of that time. Lectures are delivered regularly by the physicians, surgeons and superintendent. A good many of the nurses pass on to various posts in the Union Infirmaries throughout the country. To obtain such posts, however, it is necessary to spend six months of their training in one of these before they can get their certificate as a "Trained Nurse," passed by the Local Government Board for Ireland, which has the control of these appointments.

On this account the hospital has arranged an interchange of nurses with the Union Infirmary at Waterford, those probationers who wish to meet the requirements of the Local Government Board going for six months of their three years to the Union Infirmary, which, in exchange, sends those of its probationers who desire the greater experience of a general hospital. Probationers, at their own request, are also sent to Liverpool for a three months' course of fever training, the time being reckoned in their three years.

A premium of £15 is required before the admission of a probationer; but she receives a salary of £5 the first year, £10 the second, and £15 the third, subject to passing the examinations. She also receives out-door uniform after passing the first examination at the end of the second year, or when going to any other institution for part of her training.

Waterford, which at one time was one of the most thriving and important seaport towns of Ireland, will again look forward to a season of prosperity, now that the new route from Fishguard to Rosslare has been opened, forming a terminus of the Great Western Railway at the "Urbs Intacta" (so runs the Waterford motto). And the County and City Infirmary is doing its best to raise the standard of nursing there, as elsewhere in Ireland; the authorities look forward to the day when Irish nurses will not need to come to London training-schools to "finish" their professional education.

### Our Mortuary.

It is a restful little place, our hospital mortuary, or chapel as we call it, standing away from the big building across a yard, and nestling in some green foliage that once formed part of an old garden.

On one side of the high wall is the busy city life, overflowing with wakefulness—on the other, quiet sleep.

Immediately over the stone porch is carved an angel with outstretched arms, and above it the words, "It is well with the child," for ours is a children's hospital, therefore a children's mortuary. There at the back is a small stained-glass window representing the Good Shepherd with His flock, and underneath is an altar, which has upon it a brass cross and pair of vases, while from the centre of the roof hangs a wrought-iron lamp, kept lighted all night when the place holds a little inmate. Running round the walls are painted scrolls bearing texts—"Suffer little children to come unto Me, for of such is the Kingdom of Heaven" and "So He giveth His beloved sleep."

Six bedsteads of wrought iron are ranged, three on either side, leaving a passage down the middle of the mortuary. Each bedstead is fitted with a celluloid mattress or tray, and has a pillow with fine linen case, on which are embroidered in white silk the letters I.H.S., as also on the linen coverlets. The window, scrolls, and beds are all the gifts of a wealthy patroness, in memory of three children she lost.

A nurse is coming along now, with a small burden in her arms, covered with a sheet, and over the sheet the red blanket kept for that purpose. She is followed by a probationer with the key, who unlocks the ponderous door, and they both enter. When the coverlet has been taken from the bed and the sheet gently removed and laid neatly on, the little body is reverently put down, and nurse smooths the fair hair and folds the waxen, baby hands. A quiet whisper sends the probationer back across the asphalted yard into the hospital buildings, and she returns with some sprigs of white jessamine in her hand. A look of approval

OUR MORTUARY—*continued.*

from nurse, and the flowers are placed on the still breast and within the small fingers, and they both pause a minute before arranging the coverlet to look down on the peaceful little face, with its quaint look of age on the pinched features. He was only in hospital for two days, and, judging from the family history gleaned by Sister, this is his first restful sleep since he entered this world.

A paper with his name and age is pinned to the coverlet, and nurse and the probationer depart, the former to return again later with another still form, for the Angel Death has been busy in the wards again. It is a child of nine, this time, judging from her size, and both nurses, when their duties are performed, seem loth to leave her there. Very pretty she is, with masses of nutbrown hair and long dark lashes resting on the marble cheeks. "Heart," says nurse softly to the out-patients' nurse who has spared a few moments from her work to help her, and her eyes fill with tears, for Jessie's nine weeks of patient suffering had endeared her very much to nurse.

Later on in the day baby's parents came to see their little one before they fetched him to his last resting-place, and great was their surprise and awe when they stepped inside the mortuary. They looked more like tramps than ordinary poor people, and their clothes were absolutely in rags. The poor mother, who had been sobbing violently all the way across the court, stopped crying and held her breath as the cambric square was removed from baby's face and the coverlet turned down halfway, and she took in with wondering gaze the utter purity and peace of everything.

She touched the pillow with the tip of her finger, and said "Eh, well, eh, well" below her breath, while the man murmured, "A never thowt ter see th'like; it's grand," and then, turning to his wife, he said sadly, "We could never ha' made 'im like that." She wept bitterly then, exclaiming, "Oh, nurse, ain't he beautiful!" adding with unconscious pathos, "I never thought he could ha' looked as clean and pretty as that." She would not kiss him. "I should soil him," she said; "he don't seem like our little Joey; he's too beautiful for us to touch; it makes me right glad he ain't alive when I see him looking so lovely lying there." The man gingerly touched a strand of the fair hair and muttered again, "A never thowt ter see th'like." Before they left the mother drew out from her shawl a crushed and faded bunch of wallflowers, which she prepared furtively to throw away. "I brought them for *him*," she said, in answer to nurse's look of inquiry. "Me and his father did, but they're not good enough now," and no amount of persuasion could induce her to leave them with Joey.

Poor parents!—or perhaps happy rather. It was no longer their ugly, dirty little baby, always fretful through ill-nourishment and neglect, but something almost too noble and grand to be flesh of their flesh that lay before them, and perhaps for the first time in his eighteen months of life,—and then alas! only in death,—did Joey make his parents' hearts swell with pride over him.

. . . . . .

The lamp burns dimly in our chapel, and the white flowers give out a sweet scent, while all around reigns that absolute stillness that is found in the chamber of death. Three beds are occupied now, and the night nurse presently turns the key and opens the door to admit herself and a young mother, who has trudged many weary miles, only to arrive too late to see her little one alive. "My baby!—my little, little baby!" she cries, and covers the cold brow with kisses. Nurse lights the candles, and their rays cast a glow on the window. "Look," she says gently, and the sorrowing mother checks her sobs as she sees the loving tenderness of the Good Shepherd's face and the tired lamb in His arms. "For of such is the Kingdom of Heaven."

### Native Nursing in Italy.

I am an Italian nurse, and have worked in Italy for eleven years, but I find it somewhat difficult to give an idea of the work here. Of course I am not speaking of the foreign nurses—English, American or German—as these mostly, if not entirely, work amongst the foreigners living here or passing through. As I live in Florence I have taken that city as an example, and I think it is fairly representative of the conditions prevailing all over the country, though I have been told that in Siena more is done than elsewhere to qualify nurses for their work by giving them lectures and instructions.

The hospitals have what are called "*Inserviente*." These are women taken from the people with so little education that many can neither read nor write. It seems almost strange that order can be kept with such a staff, but for this provision is made. All hospitals have a few religious sisters, who superintend the nurses, give out the linen, and prepare the dressings, though they take no part in the practical nursing themselves. The doctors here are accustomed to do much of the work which in other countries is done by the nursing staff, though the great surgeons have their own special nurses, who, having worked with them for some time, get to know the requirements of their master. The first dressing after an operation is done by the great man himself; after that the patient, if not a first-class paying case, is handed over to an assistant surgeon.

As to private nursing, most of that is done in Italian families by the nuns, some of whom have become very skilful through practice, all their knowledge being gained at the bedside. Training nurses in the hospitals as it is done in foreign countries is unknown here as yet. A few years ago an attempt was made in Florence and in Rome by an English lady to get permission for some of the girls of the middle classes to enter the principal hospitals, and under her they worked in the wards, and attended some lectures. The period of training was, however, too short and the hours daily too few for them to become fully trained, and there was great difficulty in enforcing discipline, the girls not being housed in the hospitals. Later some of these nurses continued their work in a nursing home where there were a few surgical and some typhoid cases, and were also sent out to private cases, where many did well.

The Italians as a rule have a natural disposition for caring for the sick; they do it gladly, are not afraid of tiring themselves, and are kind and gentle. On the other hand, they do not understand cleanliness in an English sense, nor are they very exact or reliable. Still Italian doctors who have not been abroad and seen other nurses are glad to avail themselves of the services of these women who have had only this scanty training, as many families object to the religious nurse, and the managers of many hotels raise an objection to the dress.

In one hospital here where only male patients are admitted, all the work is done by male nurses, who can also be hired for private cases. Another institution which renders assistance in times of sickness, when a woman's strength would be insufficient, is the Brotherhood of the Misericordia.

In the sanatoria and asylums the lack of education is still worse, for necessarily women taken from the field, the farm, or the kitchen lack even the rudiments which go to make nurses capable of doing good work in such trying surroundings.

There is no such movement on foot in Italy as organised district nursing; such a thing would be absolutely foreign to the peasant nature. It will take a long time for the Italians of the better classes to realise that nursing is a high calling, and one which their daughters would do well to undertake, for they are imbued with the idea that sick nursing is simply a synonym for the religious life.

### The Open-air Treatment for Diarrhœa and Vomiting.

In the summer and early autumn our hospitals and infirmaries are always crowded with babies and children suffering from diarrhœa and vomiting. Unimportant as these symptoms may sound, everyone who has had any nursing experience knows how quickly the patient may be reduced to a dangerous state of collapse, and it is not often that we are called upon to treat or nurse these patients until they are already in such a condition. A short time ago, looking over the children's wards of a large infirmary, one was impressed with the general healthy look of the little patients, most of whom were convalescing from the prevailing complaint. The sister in charge said that the usual method of treating these little ones was to nurse them in the open air as much as possible.

As soon as the child is admitted and has had the necessary hot bath, it is wrapped in a blanket and put in its cot, which, sheltered from too much wind or sun, stands on the bridge—or outdoor communication between the wards—from about 7 A.M. till dusk.

That this is a most reasonable proceeding cannot be denied, when one knows how often these poor scraps of humanity come from the most airless, stuffy, and filthy streets. This alone is often responsible for their lowered state of health, and under these favourable conditions the first prevailing disease which comes along finds a "happy hunting ground."

So from a logical, hygienic, and also pleasant point of view this treatment should be popular. The fresh air is assisted in its good work by judicious feeding with peptonised milk. Water is administered freely where there is much and frequent vomiting, repeated doses of which act as a "wash-out." Mist. ol. ricini may be given, if the patient is not too ill, during the first day.

Instead of brandy, if a stimulant is necessary, a mixture containing one minim of strychnine in each dose is given about six-hourly or as required. A bath is given night and morning.

When both symptoms have disappeared, the child is allowed to proceed to Benger's food and finally to milk puddings. Some of the merry little people trotting round had been severely ill when admitted, but they certainly showed every sign of perfect recovery and looked sunburnt and well after their excellent treatment.

### Presentations.

Ratho, Kirkliston, and Newbridge Districts.—Miss S. Sothern, who has acted as district nurse for Ratho, Kirkliston, and Newbridge for some years, having resigned her post in order to take up duties elsewhere, she has been presented by the committee with a set of brushes, mounted with silver, and a purse of sovereigns in acknowledgment of the excellent work done by her.

### Everybody's Opinion.

[Correspondence on all subjects is invited, but we cannot in any way be responsible for the opinions expressed by our correspondents. No communication can be entertained if the name and address of the correspondent are not given as a guarantee of good faith, but not necessarily for publication. All correspondents should write on one side of the paper only.]

### AMERICA AND ENGLAND.

"W. Z. B." writes: I think that "B. S. A." must have been very unfortunate during her short visit to the United States of some six months, for it is well known, I think, that, though rather rough and ready, and sometimes rather eccentric, the doctors are no more—at least the better-class men—lacking in refinement, courtesy, and consideration for their nurses, than our own very justly, highly respected and talented English medical men. If "B. S. A." had been able to spend six years instead of six months in the States, I think that she would have found reason to alter her opinion, and would not have endeavoured, apparently from spite, or from some other paltry reason (probably she was very incompetent and got snubbed, possibly justly, by the medical men) to take away the characters of a very worthy body of men and women. The Almighty dollar, no doubt, plays a more important part in America than here, but I venture to think that patience, self-sacrifice, and many other virtues are to be found among American nurses nearly, if not quite as much, as among their more favoured sisters in England. If I may venture to say so, I can only express my surprise that "B. S. A." should care to make such foolish accusations against a body of women who are, probably, very much her superiors in all that concerns the noble profession of nursing and true womanhood. It is so evident that her letter is simply a counter-attack, and a very ignorant one, apparently, to the partisan, but far more reasonable letter of an "American Nurse" some time ago, that perhaps really it is hardly worth notice.

### SISTERS AND ORDERLIES.

"Connaught Ranger" writes from North Camp, Aldershot: Under the heading "Army Sisters and Orderlies" "Aubrey" endeavours to express an opinion on the relations which exist between the female staff and the male of military hospitals. "Aubrey's" opinion seems based on the fact that he has met several Army sisters. I trust you will allow me to express my views as one who has served under them for four years and am still in the same unenviable position. Personally, I have nothing to be grateful for, as previously to the Army sisters taking complete charge of the wards I had absolutely a clean record; but, for some undefined reason, I soon got in trouble, thanks to the kindly assistance of Army sisters and nurses, who are so kind to the orderlies of the Royal Army Medical Corps. I may point out that I am very plain spoken, and perhaps this is what may have caused these ladies to take a violent dislike to me. I can furnish further cases similar to my own in the proportion of 70 out of 100 orderlies who have likewise for no apparent reason been treated the same. "Aubrey" quotes the National Hospital, Bloomsbury, as an example of the frictionless cooperation existing between the male and female staff of that institution. There is no comparison between the two. The worst terror for a man in the National Hospital who misconducts himself or who is inefficient is dismissal. In a military hospital an orderly is subjected to all the petty tyrannies imaginable by reason of the unusual authority given to the sisters and nurses, who are not too scrupulous as to the manner in which they use this authority, such diverting things as bringing trivial charges against the men under them, imposing vexatious tasks, refusing reasonable requests, being every day matters, etc. I may add that the majority of medical officers in the Army Medical Corps consider it only the duty of a gentleman to believe the sisters and nurses and to ignore the orderly's reply; consequently they punish men severely, believing that the man concerned has endeavoured to take advantage of the weaker sex. When a fresh commanding officer came to this station the first punishment he awarded (imprisonment) was to a youth of nineteen years of age for a so-called charge of insolence to a nursing sister, and this by one of the best commanding officers (apart from the feminine influence) a man could possibly serve under. Again, three years ago a sister was here who averaged more courts-martial than any three officers in the corps. I mean that she only was the direct cause, and no other. I commend "Aubrey," who, I think, is a little effeminate, to confine himself to his own circle, and not presume on the score of having met a few Army sisters to consider that he has any right to discuss or criticise the Army Medical Corps, who are, I am sure, just as chivalrous and respectful to a lady whatever profession she may belong to, as he himself may find, if he cares to use his powers of observation a little more keenly when he happens upon any of this corps. I maintain that men will not join the "Nursing Section" except under compulsion by reason of the unfair treatment meted out to those who have incurred the dislike of one sister or the other. There are very few good, just sisters or nurses; they average about one in each first-class military hospital.

### THE DISMISSAL OF A SUPERINTENDENT NURSE.

"Crusader" writes: A bare sense of justice leads me to comment on the recent unfortunate happenings at the Brentford Isolation Hospital. In the first place, the superintendent nurse is reported to have been dismissed. It is difficult to see how a superintendent nurse can be held responsible for the admission of patients into any hospital, or for deciding upon the wards in which they shall be placed. Such matters are usually decided by the medical officer in charge. Secondly, in the present state of the smaller isolation hospitals it is practically impossible to prevent the occasional occurrence of such unfortunate incidents. A typical hospital has four wards, say, male and female enteric, and male and female scarlatina. Supposing all the wards to be occupied and a patient in the scarlet wards develops measles, what is to be done with it? Sending it home is out of the question, and there it has to remain, a source of danger to the rest of the patients. The worried medical officer and matron turn the wards upside down and generally move heaven and earth to get the case isolated if possible, sometimes only to find, about ten days later, one or two fresh cases cropping up. And so the thing goes on. Obviously, no new patients should be admitted until the epidemic is stamped out and the wards have been thoroughly cleansed and disinfected. But this is easier said than done in the case of a hospital serving a district of, possibly, 30,000 inhabitants, with sporadic cases of scarlatina occurring all up and down the district. First cases must be isolated, or an epidemic will ensue and it is a serious matter to close the wards of the only available hospital for two or three months. In a case that came under my observation there were 22 children suffering from scarlatina in two wards. A child who had been in the hospital two months developed measles. There happened to be an empty ward available and she was immediately isolated. Ten days later a second case occurred; again, twelve days later two fresh cases, and so on until altogether eight children were down with measles, the last case having the rash out exactly six weeks after the first one. In another case there were 14 cases of scarlatina in a ward. Of these cases one developed measles two days after admission; a second developed chicken-pox the third day after admission; while yet a third child was discovered to have whooping-cough. The measles case was immediately isolated, but the unfortunate whooping-cough child (who was, of course, recovering from her attack of scarlatina) also developed measles! So the ward presented the unusual spectacle of scarlatina, measles, chicken-pox and whooping-cough all mixed up together, and continued to do so, for there were no facilities for isolation, the two remaining wards in the hospital being occupied by enteric cases. Fortunately, all the cases did well and the wards were ultimately disinfected without further mishap. The whole question is an urgent one. Certain hospitals appear to have solved it by means of the cubicle system, *i.e.* the complete separation of each patient, from the beginning. It would be interesting to hear how the system works where it has been given a fair trial.

### "AN INDICTMENT OF THE MIDWIVES ACT."

"W." writes: I venture to think that an indictment of the Midwives Act which appeared in The Nursing Mirror of September 15 might more fairly and fitly be entitled an indictment of the lack of public responsibility. To quote from The Hospital of the same date, in a paper on "Medical Opinion" (page 418) you rightly press home to the public how great is their share of blame in the case in the absence of interest shown in asylums, which they continue to treat as "dustheap products." Well, the management of poor mothers and infants is relegated to the same position, and the Midwives Act cannot be fairly blamed for failing to awaken what is a great necessity—the State holding aloof as it does—an active living interest in a subject of vast importance to the future of our race. The interesting letter contributed by an inspector of midwives and published in the same issue only accentuates a series of facts which were well known to those who pressed forward legislation; the figures now available only strengthen their contention that the bulk of confinement cases among the poor —60 to 70 per cent.—were in the hands of ignorant, illiterate, and often extremely dirty women. That hundreds of these women have retired from practice since the Act came into force again further strengthens the argument that a better class is required, a more intelligent and cleaner type of woman.

The Inspector writes from her own experience, but the figures relating to English and Welsh midwives show how enormous is the proportion of untrained women still practising. Let me submit these figures: there are on the Midwives Roll over 20,000 names, but of these only 12,255 are practising midwives, and out of that number 8,115 are untrained! The Act did not create this condition; its provisions should, if properly used by local authorities, gradually remove from the Roll the names of women who are actively dangerous by reason of incompetence, drunken habits, or general malpractice. But no power is given by the Act to any public body to create a new race of clean, competent women, who should be ready to replace the unfit as they retire. Had such a scheme, however, been included in the Act, it would have been, I fear, bitterly resented; therefore, as feudal England, for good or for ill, must respect the "vested interests" of even an incompetent midwife, and while doing so elects to provide no machinery by which her place in due time may be taken by a more enlightened type, the appeal must be to a sense of public responsibility in this matter, which appears to a large extent to be non-existent. This question is one that cannot receive full consideration by such inadequate means as a paragraph dealing with one side only, namely, the small earnings of practising midwives. That fact had been fully realised for years by those who promoted legislation, but they rightly failed to see in it a sufficient cause for our country to remain in an attitude of careless passivity unknown to other lands regarding the well-being of its poorer mothers. The working of the Act has simply brought into fuller light the following facts that (1) a large number of midwives are untrained, and are often dangerously ignorant; (2) that the calling is an ill-paid and unpopular one; (3) that the public, although they speak and write much of the increase of terrible infant mortality, are utterly uninterested in the ways in which an infant is given its first start in life—of all questions one of the most pressing. There are other points that the Act is making clear, while they certainly do not show that midwifery is a popular calling. In some rural districts doctors now charge a double fee when called in to cases in which a midwife is employed. This course is seldom taken in towns where doctors do not find the low fee available from the working classes sufficient to meet the serious drawbacks of the inevitably broken nights involved in large midwifery practice, with a heavy round of visits next day to general cases. I state this as a fact without any comment on the medical point of view, which is doubtless natural enough. As a fact, however, this attitude will not make the work of a midwife financially more attractive, as her fee is usually 5s. to 7s. 6d., and her cases are not likely to increase when a double fee to a doctor is a possibility. What, we may ask, would be the amount of nursing of our sick poor in their own homes be if no large sum—£70,000—had not been given for its improvement by the late Queen? And what would that sum represent if the public had not loyally and generously added to it year by year? Till those bodies which train and place midwives are more heartily supported there will be a dearth of these workers; for training and a sum to ensure their support for one year at least are absolutely needed. If this sum were insured a woman could often add to her sparse midwifery cases some other calling, as is done constantly abroad; but for the first year at least she should have a definite sum assured to her besides the fees from cases. She has no capital, and has often a family to support. The Association for promoting the training and supply of midwives has as patron the Queen, and for president the Archbishop of Canterbury, but in spite of this support and of many appeals and well-planned meetings to explain its object, the funds of the Association are low, and the training may have to be curtailed in the future. This is but one of many private bodies that are thwarted by lack of funds and interest for the furtherance of a most needful work. It should, however, be remembered that the Midwives Act was passed with the object of reducing maternal and infant mortality, and that if the public remain passive and uninterested in its success, and the number of midwives is greatly reduced, the Act will have in time saved many mothers from incompetent treatment, by gradually removing from responsibility women who were and are totally unfit for its exercise. The question of fees and how far the poor can adequately meet those which a medical attendant considers are the lowest he can fairly charge may then be considered on a wider basis than hitherto; further, the system practised in Ireland since 1851, by which the Local Government employ a large staff of parish midwives, whose services are free all over the country, may also receive the attention it deserves, as one of many ways of meeting a real need.

### Midwifery Appointment.

Queen Charlotte's Hospital.—Miss Cherry R. Lilly has been appointed assistant night midwife and staff nurse on Queen Charlotte's Notting Hill and Kensington districts. She was trained at Queen Charlotte's Lying-in Hospital, and previous to her present appointment had done district and private nursing in England and Wales. She has also acted locum tenens for a Queen Charlotte's Hospital district in 1904, and taken holiday duty with entire charge of a private nursing home. She holds the certificate of the Central Midwives Board.

### Novelties for Nurses.

(By our Shopping Correspondent.)

### INVENTION BY A NURSE.

A new bed-pan and bed-bath combined has been invented by a nurse, and, according to high authorities on the subject, it is likely to supply a distinct want and command a ready sale. It has been provisionally patented for six months, but the nurse, having been obliged to give up work owing to a breakdown in health, does not feel in a position to go on with the business part of the invention herself, and would be glad to hear from anyone who would care to purchase the idea. Her address is Nurse Bryan, 5 Ellenborough Crescent, Weston-super-Mare.

### To Nurses.

We invite contributions from any of our readers, and shall be glad to pay for "Notes on News from the Nursing World," "Incidents in a Nurse's Life," or for articles describing nursing experiences at home or abroad dealing with any nursing question from an original point of view, according to length. The minimum payment is 5s. Contributions on topical subjects are specially welcome. Notices of appointments, letters, entertainments, presentations, and deaths are not paid for, but we are always glad to receive them. All rejected manuscripts are returned in due course, and all payments for manuscripts used are made as early as possible after the beginning of each quarter.

### Appointments.

[No charge is made for announcements under this head, and we are always glad to receive and publish appointments. The information, to insure accuracy, should be sent from the nurses themselves, and we cannot undertake to correct official announcements which may happen to be inaccurate. It is essential that in all cases the school of training should be given.]

Achil Hill Sanatorium, Milnathort, N.B.—Miss Margaret C. Robertson has been appointed staff nurse. She was trained at the Victoria Infirmary, Glasgow, and has since done private nursing.

Braintree Union Infirmary.—Miss M. A. Newns has been appointed superintendent nurse. She was trained at the Rochdale Union Infirmary, where she afterwards became charge nurse and night superintendent. She holds the certificate of the Central Midwives Board.

Florence Nightingale Hospital, Bury, Lancashire.—Miss Mary A. McGuirk has been appointed sister. She was trained at the Leith Hospital and at the Belvidere Fever Hospital, Glasgow.

Ilford Isolation Hospital, Chadwell, Essex.—Miss I. Millar has been appointed night superintendent. She was trained at the Fleming Memorial Hospital, Newcastle-on-Tyne. She has since been staff and subsequently charge nurse at the Park Hill Hospital, Liverpool, and sister at the Borough Sanatorium, St. Helen's, Lancashire.

Isolation Hospital, Norwich.—Miss M. Beatrice Vickers has been appointed assistant matron. She was trained at the Royal Infirmary, Bradford, where she was afterwards sister of the Gynæcological wards. She has since been sister at the Bradford Children's Hospital; night superintendent at the City Hospital for Infectious Diseases, Newcastle-on-Tyne; and night sister at the Jenny Lind Infirmary, Norwich.

Isolation Hospital, West Bromwich.—Miss A. Davis has been appointed matron. She was trained at the Monsall Fever Hospital, Manchester, and at St. George's Hospital, London. She has since been assistant matron at the Isolation Hospital, Norwich, charge nurse and night superintendent at the North-Eastern Hospital, South Tottenham.

King Edward VII. Sanatorium, Midhurst.—Miss Ethel Bentley has been appointed sister. She was trained at Hope Hospital, Manchester, where she was afterwards sister, and has since been sister at the Heswall Sanatorium, Cheshire.

Lincolnshire County Nursing Association.—Miss Frances Gibson has been appointed assistant-superintendent. She was trained at the Royal Infirmary, Liverpool, and at the Ladies' Charity and Lying-in Hospital, Liverpool. She has been Queen's Nurse in Liverpool and at Woolton, and has also done private nursing.

St. Monica's Hospital, Easingwold.—Miss F. E. Smith has been appointed matron. She was trained at Bethnal Green Infirmary, and for fever training at Bagthorpe Infirmary, Notts, and at Dartford, Kent. She has since been sister at Huddersfield Infirmary, and at Sparrow's Nest, Sheffield, and has also done private and district nursing in connection with the Victoria Nurses' Home, Ipswich.

Stepping Hill Hospital, Stockport.—Miss Katherine Burman has been appointed night superintendent. She was trained at the Brownlow Hill Infirmary, Liverpool, and has since been superintendent nurse at the Poor-law Infirmary, Totnes, and at Caistor Infirmary. She holds the certificate of the Central Midwives Board.

### Notes and Queries.

**REGULATIONS.**

**The Editor is always willing to answer in this column, without any fee, all reasonable questions, as soon as possible.**

**But the following rules must be carefully observed.**

1. **Every communication must be accompanied by the name and address of the writer.**
2. **The question must always bear upon nursing, directly or indirectly.**

**If an answer is required by letter a fee of half-a-crown must be enclosed with the note containing the inquiry.**

*Broken Engagement.*

(286) I am engaged with a permanent case, the patient dies, and I am only paid until death.—*Puzzled.*

Everything depends upon the terms of the engagement. If you are engaged yearly, you are entitled to payment in lieu of notice. If you are paid monthly and engaged by the month, you receive a month's salary in lieu of notice. If you are paid weekly you receive a week's salary in lieu of notice.

*American Journal.*

(287) Where can I get an American nursing paper?—*Inquirer.*

If you write to the Scientific Press, 28 Southampton Street, Strand, the manager will get you an American journal of nursing.

*Epilepsy.*

(288) Can you tell me of a home in Yorkshire where an epileptic boy could be received and taught to earn his living? *Paddock.*

Write for advice to the National Society for Employment of Epileptics, Denison House, Vauxhall Bridge Road, Westminster, London, and to the David Lewis Manchester Epileptic Colony, Warford, near Alderley Edge, Cheshire.

*Missionary Nursing.*

(289) Will you tell me where I can hear about missionary nursing in connection with the work?—*V. T.*

The Church Army, 130 Edgware Road; The Church Missionary Society, 16 Salisbury Square, London, E.C.; Edinburgh Medical Missionary Society, 56 George Square, Edinburgh; and the Nurses' Missionary League, Miss K. Millar, 28 Queenborough Terrace, Bayswater, W.

*Nursing Home.*

(290) Can you give me the address of Miss War, who keeps a nursing home in London?—*Star.*

We do not give private addresses. Perhaps our readers can help, and if they enclose stamp will forward the answer.

*Sanitary Inspectorships.*

(291) Where can I get information respecting factory inspectorships and certificates required?—*Chesterfield.*

Write to the Sanitary Institute, Margaret Street, W., and to the Sanitary Inspector Examination Board, 1 Adelaide Buildings, London Bridge, E.C.

*Medico-Psychological Medal.*

(292) How can I obtain another medal? I have lost mine.—*Lombardi.*

The only way is to write to the Association and explain your difficulty.

*Epilepsy.*

(293) Where is there a home where a girl of 23, who is epileptic, can be received? We can only afford from 8 to 10 shillings weekly.—*Kate.*

The Meath Home of Comfort, at Godalming, is the best place for her. The terms are a little higher, but possibly an arrangement might be made. The Liverpool Home for Epileptics, at Maghull, near Liverpool, is lower in terms; and the Homes of the National Society for the Employment of Epileptics, 12 Buckingham Street, Strand, are also suitable.

*Nurses' Missionary Association.*

(294) Can you tell me the address of the Nurses' Missionary Association quarters?—*Marylebone.*

Address Miss K. Millar, 28 Queenborough Terrace, Bayswater, W.

**Handbooks for Nurses.**

| | Post Free. |
|---|---|
| "A Handbook for Nurses." (Dr. J. K. Watson) ... | 5s. 4d. |
| "Nurses' Pronouncing Dictionary of Medical Terms" | 2s. 0d. |
| "Art of Massage." (Creighton Hale) ... ... ... | 6s. 0d. |
| "Surgical Bandaging and Dressings." (Johnson Smith.) ... ... ... | 2s. 0d. |
| "Hints on Tropical Fevers." (Sister Pollard.) ... | 1s. 8d. |

Of all booksellers or of The Scientific Press, Limited, 28 & 29 Southampton Street, Strand, London, W.C.

### For Reading to the Sick.

SUNDAY IN SICKNESS.

Slowly through the twilight grey,
Dawns again the Sabbath Day;
Once again the bells are ringing,
From the quiet valley bringing
Invitation sweet to me,
To look up and worship Thee.

They who in communion here,
With the holy and the dear,
Thrilling anthems loudly raise,
To Thy glory and Thy praise—
Not more blessed are they than she
Who is left alone with Thee.

Others in Thy house of prayer
Seek Thee, and they find Thee there;
In my lowly chamber, I
Worship Thee adoringly;
Graciously Thou blessest me,
Bidding me look up to Thee.

*Anon.*

Always when we pray to be guided we must take God's way wherever it may lead us; we must let God decide whether we shall work or rest. One writes: "No time of seeming inactivity is laid upon you by God without a just reason. It is God calling upon you to do His business by ripening in quiet all your powers for some high sphere of activity which is about to be opened to you." We are doing God's work not only when we are pressing forward in eager haste to accomplish some achievement for Him, but quite as much when we are keeping still and allowing God to work in us, enriching and beautifying our lives. "They serve the Lord who only stand and wait." We are in this world not only to do a great deal of good, but also to grow into the likeness of Christ. If then in any certain weeks we are not permitted to do any kindnesses, but if, meanwhile, we have been growing a little more patient, gentle, thoughtful, humble, if the peace of our hearts has become a little deeper, quieter, sweeter, the time has not been lost. We need then to have God's direction, or we shall perish. In the darkest hour of Christ's life, when He could not see even His Father's face, and cried out like one forsaken, He still kept His faith in God firm and strong. It was still, "My God, my God." But while there are times when we need guidance in an unusual way, there is no day in all our brightest year when we do not need it, when we dare to go forward one step without it.—*J. R. Miller, D.D.*

To us the path of each day is always new—we have not passed this way heretofore, and we cannot tell what any hour may bring to us. But He knows all the way, for He went over every inch of it. There is no human experience which Christ does not understand. No suffering can be ours which He did not feel. No wrong can hurt us, but He was hurt far more sorely. Is the burden heavy? His burden was infinitely heavier, for He took our infirmities and bore our sicknesses, and bowed beneath the load of our sins. There is no phase of struggle, of suffering, of pain, of temptation, or of joy, with which He is unacquainted.

There is no secret sigh we breathe
But meets the ear divine.
And every cross grows light beneath
The shadow, Lord, of Thine.

*Anon.*

